# Detection of EEG Activity in Response to the Surrounding Environment: A Neuro-Architecture Study

**DOI:** 10.3390/brainsci15101103

**Published:** 2025-10-14

**Authors:** Jesús S. García-Salinas, Anna Wróblewska, Michal T. Kucewicz

**Affiliations:** 1 Brain and Mind Electrophysiology Laboratory, Multimedia Systems Department, Faculty of Electronics, Telecommunications and Informatics, BioTechMed Center, Gdansk University of Technology, 80-222 Gdansk, Poland; anna.wroblewska@pg.edu.pl (A.W.); michal.kucewicz@pg.edu.pl (M.T.K.); 2 Department of Environmental Design, Faculty of Architecture, Gdansk University of Technology, 80-222 Gdansk, Poland; 3 Department of Physiology and Biomedical Engineering, Mayo Clinic, Rochester, MN 55905, USA; 4 Department of Neurology, Mayo Clinic, Rochester, MN 55905, USA

**Keywords:** EEG, neuro-architecture, biophilic environments

## Abstract

**Background**: The external environment continuously shapes human perception, often without conscious awareness. This influence can be objectively studied using non-invasive recordings of brain activity in controlled virtual reality (VR) settings. We hypothesized that brief presentations of natural features would elicit distinct neural oscillatory patterns detectable through changes in the spectral power of resting-state electroencephalogram (EEG) activity in specific brain regions. **Methods**: To test this, participants passively viewed three minimalist VR environments—with and without biophilic elements—while their EEG was recorded. **Results**: Our results revealed consistent changes in spectral power, particularly suppression in the alpha band and an increase in the theta band in the occipital cortex. **Conclusions**: These findings support the use of resting-state EEGs in immersive VR as a promising and objective method for evaluating how specific design elements influence neural activity, offering valuable insights into the field of neuro-architecture.

## 1. Introduction

The human brain is in a continuous interaction with its surrounding environment, regardless of whether we are aware of it. An immense array of external stimuli affect thoughts, emotional states, and behaviors [1]. The aspects of our surroundings, designed and constructed by humans are known as the built environment, where we spend up to 87% of our lifetime [2], with little conscious awareness of its particular elements. Studying neural responses to such subconscious external stimuli can have a profound impact on architectural design and our understanding of the visual perception of spaces.

A key dimension of this interaction involves our intrinsic connection to nature. According to biophilic theory, humans are biologically predisposed to require contact with natural elements [3]. Hence, a well-designed built environment should fulfill this fundamental need, which originates from our evolutionary history and adaptation to the natural world [4,5]. When urban designs lack this connection, they can evoke solastalgia, a distressing feeling caused by the direct experience of the negative transformation or desolation of the physical environment [6,7]. The absence of natural elements in the environment has been shown to have a profound impact on well-being and emotions [8,9,10]. It has been reported that patients in hospital rooms with a view of natural surroundings not only recover faster but also require less pain medication and experience fewer post-operative complications [11]. A broad analysis of hospital rooms found that certain architectural design elements, including natural features such as bedside views of nature or images on screens and increased daylight, can alleviate patient stress and pain while decreasing the hospital stay duration [12]. Stress Reduction Theory proposes that exposure to natural environments has a stress-lowering effect [13,14,15,16,17,18,19,20]. Moreover, Attention Restoration Theory proposes that natural elements effortlessly and involuntarily attract attention, thereby restoring cognitive functions and reducing stress [15,21,22,23]. Through questionnaire research and the measurement of subjective feelings, other studies have found a relationship between physical space, the proportions of corridors in buildings, and emotional responses [24].

Neuro-architecture is an emerging approach that explores the interaction between the environment and brain activity, and it has been used in previous studies to identify neural correlates of viewing photographs of natural scenes in forests or urban greenery [25,26,27,28]. A more systematic and immersive approach proposes the use of virtual reality (VR), which enables the investigation of neural responses to built environments with and without natural elements. Unlike static images, VR allows dynamic interaction with the environment and provides greater experimental control over environmental stimuli. The inclusion of immersive VR enhances the ability to study environmental effects in a controlled, yet highly realistic, manner that is able to induce an emotional response [29,30,31,32,33]. With the combination of EEGs and VR, our approach attempts to capture an objective measure of the neural activity associated with these psychological theories, bridging subjective reports with physiological data. By simulating built environments with different elements, researchers can systematically analyze the cognitive and emotional effects of particular architectural elements and features. This multidisciplinary approach bridges the gap between neuroscience and architecture, fostering the development of evidence-based design strategies aimed at improving human well-being.

Recent advancements in computational neuroscience have enabled the extraction of complex patterns from EEG data, thereby providing deeper insights into the neural mechanisms associated with architectural perception. The integration of physiological measures such as heart rate variability and skin conductance responses complements neuroimaging findings, offering a holistic perspective on how built environments influence mental states. By adopting an interdisciplinary approach that incorporates psychology, architecture, and neuroscience, neuro-architectural research has the potential to redefine how spaces should be designed to have a positive effect on cognitive function, mood, and overall quality of life.

Previous research on the effects of the natural environment has typically relied on questionnaires, indirect behavioral responses, or general outcomes, such as patient recovery time [11,12,34,35,36,37,38]. However, a psychological and physiological approach measuring responses to green walls in VR scenarios [31] concluded that such elements could reduce anxiety, with observed increases in alpha activity in the parietal and occipital lobes. Notably, they found that excessively large green walls may have the opposite effect, producing alpha decreases and stress in the participants. Therefore, similar elements produce different responses according to their features. In addition, visual stimulation from plants has also been associated with increased relative theta power in the occipital lobe [39], a response that has been linked to both enhanced visual processing and relaxation states.

Based on these findings, this study focused on the detection of neurophysiological changes produced by green elements in virtual architectural spaces using EEGs. We hypothesized that brief presentations of natural features can elicit distinct neural oscillatory patterns detectable through simple EEG analyses. This suggests that straightforward neurophysiological assessments can provide objective criteria to inform early-stage design decisions. Furthermore, we expect these effects to vary depending on the scale and integration of green elements within the space. By confirming these neurophysiological responses in a controlled VR environment, our work supports the use of immersive evaluations as a practical tool for architects, urban planners, and designers to assess the cognitive and emotional impacts of natural elements prior to construction.

## 2. Methods

### 2.1. Participants

The study involved 22 volunteer participants, 10 males and 12 females, with a mean age and standard deviation (SD) of 28.70 ± 4.04. All participants were healthy adults with no history of neurological or psychiatric brain disorders. Informed consent was obtained from all participants, ethical approval was provided by the local ethics council at the Gdansk University of Technology, and the research was performed in accordance with relevant guidelines established in the Declaration of Helsinki. Exclusion criteria were considered, such as consumption of caffeinated beverages or any medication, which could potentially affect the electroencephalography signals.

### 2.2. Experimental Procedure

The experiments were evenly distributed across different hours of the day (10 a.m.–10 p.m.), with participants performing at different times. All sessions were conducted in a dedicated room. The participants were required to report any use of corrective eyewear to ensure optimal visual perception of the virtual environment. The use of eyeglasses was allowed, as the headgear accommodated them seamlessly without compromising the equipment setup or fidelity of the displayed virtual environment. To minimize artifacts in the EEG recordings, participants were instructed to remain seated and refrain from movement. After an adaptation period to the VR equipment of approximately 3 min, we confirmed with each participant that none of them reported symptoms of motion sickness while wearing the virtual reality goggles.

**Figure 1:**
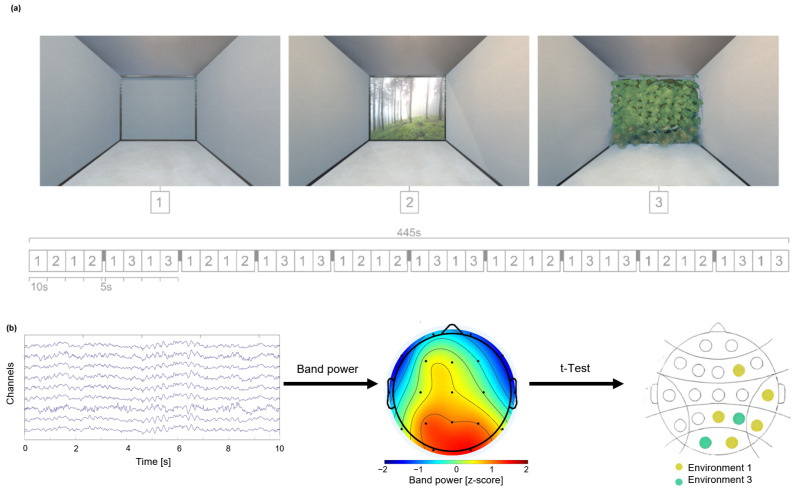
Experimental procedure. (**a**) Participants experienced three virtual environments presented sequentially, each varying in the presence of biophilic elements. The experimental protocol consisted of alternating 10-s exposures to the different environments, separated by 5-s black screen intervals, for a total duration of 445 s (7.4 min). Participants remained stationary and engaged in passive viewing, with no tasks assigned during the session. (**b**) Example pre-processed EEG signals are shown during a 10-s segment. Signals were transformed to the frequency domain by extracting band power. Later on, a comparison across environments using a signed rank test revealed significant differences in specific electrodes.

We analyzed three environments that shared a common structure and differed only in specific elements of the design or their lack (Figure 1a). They were presented through virtual reality technology while participants were sitting in a chair in a 3 m × 2.5 m × 4 m (width × height × length) room. The virtual environments were designed to have similar measurements as the real room. Environment 1 (Env1) had a gray-scale color of the base elements of the ceiling, floor, and walls, and it was free of any other elements. After immersion in Env1, the features of the virtual environment were changed. In Environment 2 (Env2), there was a window with a forest view that appeared in front of the participant, and in Environment 3 (Env3), a wall with plants was presented, which represented indoor greenery.

The procedure involved repeatedly changing the environment every 10 s, starting with alternating between Env1 and 2, or Env1 and 3, followed by a 5-s break with a black screen (Figure 1a). The procedure focused on stationary perception over exploration; no specific task was required for the participants to do; only the perceptual response to the architectural elements was the object of study. Different virtual environments were presented, with the head facing straight and with no lateral movements. The total duration of the experiment was 445 s. All results are presented as mean ± S.E.M. unless stated otherwise.

### 2.3. Equipment and Software

The experiment was conducted in a virtual reality (VR) environment designed to provide a highly immersive experience. Participants interacted with the VR environment using the Oculus Quest (Meta) headset with a resolution of 1832 × 1920 pixels per eye and a refresh rate of 90 Hz, ensuring smooth and realistic visual rendering. The research environment was developed in the Unity Engine, allowing precise control over experimental conditions and stimulus presentation.

**Figure A1:**
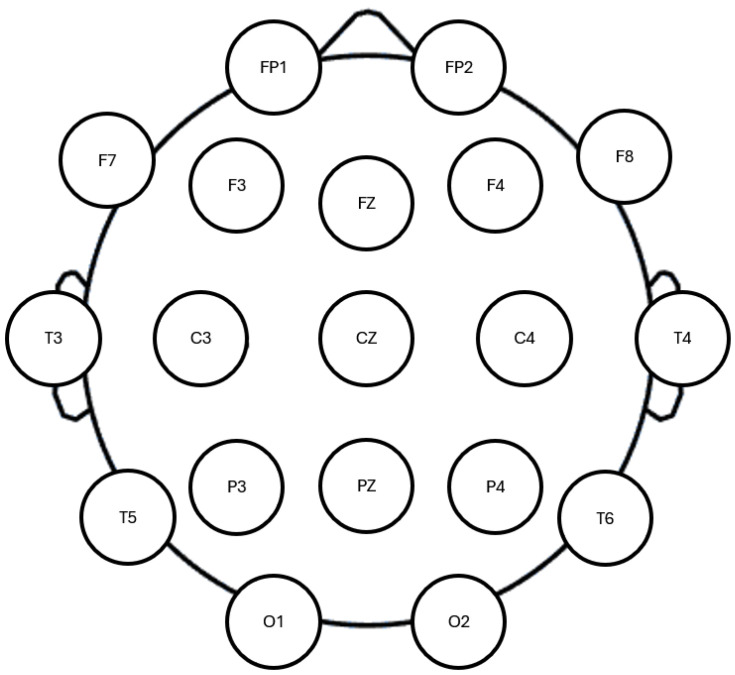
EEG electrode placement. Diagram showing the distribution of the 19 wet electrodes positioned according to the international 10–20 system.

EEG signals were recorded using a 19-channel eWave-24 Science Beam EEG system at a sampling rate of 500 Hz. Wet electrodes with conductive gel were placed according to the 10–20 international system at the following scalp locations: FP1, FP2, F3, F4, C3, C4, P3, P4, O1, O2, F7, F8, T3, T4, T5, T6, FZ, CZ, and PZ (Figure A1). A linked ear reference was used to minimize potential reference noise. Data pre-processing and analysis were performed in MATLAB 2023a (MathWorks Inc., Natick, MA, USA) using the EEGLAB toolbox [40]. Figure 1b illustrates a 10-s example after artifact removal, and the subsequent feature extraction process.

Finite Impulse Response (FIR) band-pass filtering was applied to all 19 EEG channels, restricting the signal bandwidth to 1–48 Hz. This pre-processing step effectively removed low-frequency drifts and high-frequency noise, including the 50 Hz electrical line interference. Electrodes that exhibited malfunctions or disconnections during recording, an average and SD of 0.9 ± 0.86, were removed, and their signal was interpolated.

To enhance the signal quality, Independent Component Analysis (ICA) was applied to decompose the EEG data, followed by an automatic artifact detection method called Iclabel [41]. Components associated with muscle activity, eye movements, and cardiac signals were identified based on their spatial and spectral characteristics and subsequently removed, with an average and SD of 5.72 ± 1.9 rejected components. Additionally, visual inspection was conducted to ensure proper artifact removal.

The pre-processed signals were then segmented into 10-s trials. For each trial, the average power was computed within distinct EEG frequency bands: delta (1–4 Hz), theta (4–8 Hz), alpha (8–12 Hz), low beta (12–20 Hz), high beta (20–30 Hz), low gamma (30–39 Hz), and high gamma (39–48 Hz). Figure 1b shows an example of a topological map. These standard frequency ranges were chosen specifically for the study of cognitive functions and underlying neural activity [42], and the beta and gamma bands were subdivided to obtain a higher spectral resolution. While time–frequency methods can provide a more detailed characterization of non-stationary neural signals, the band power approach was selected in this study due to its simplicity, and its ability to facilitate comparability with previous EEG literature.

To account for inter-subject variability, the power values were normalized into z-scores across all electrodes within each frequency band, as defined in Equation (1).

(1) $$Z_{i,f}=\frac{X_{i,f}-\mu_{f}}{\sigma_{f}}$$

where $Z_{i,f}$ is the normalized power of electrode *i* in frequency band *f*, $X_{i,f}$ is the band power, $\mu_{f}$ is the mean band power, and $\sigma_{f}$ is the standard deviation of the band power.

### 2.4. Statistical Analysis

Statistical analyses were performed using MATLAB 2023a software (MathWorks Inc.). To identify significant differences in neural activity across the environments, we used the Wilcoxon signed rank test (alpha threshold of 0.05) for each electrode and frequency band. Samples from the compared environments were paired for each participant (N = 22). The resulting values were visualized as topographical maps, remarking the electrodes with significant differences for each frequency band. To control for multiple comparisons, the Bonferroni correction was applied to the *p*-values obtained for each frequency band. The minimum detectable effect size (Cohen’s dz) at 80% power was estimated to be approximately 0.34, indicating that the study is sensitive to moderate or larger differences between environments.

## 3. Results

**Figure 2:**
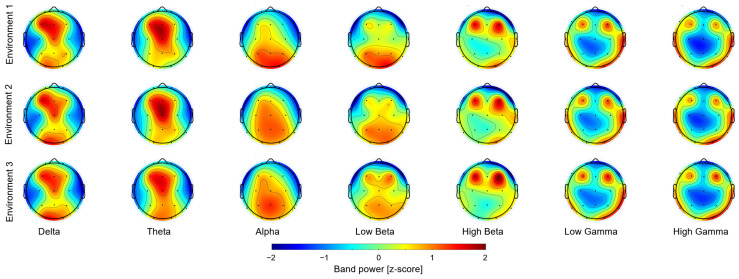
Distinct band power patterns emerge in lower- and higher-frequency bands. Low- frequency bands exhibit higher power in the central frontal than the occipital regions, whereas high-frequency bands show increased power in lateral frontal, temporal, and parietal areas. Although this pattern was consistent across environments, statistical analysis (Figure 4) revealed significant differences.

To identify specific EEG activity changes in response to biophilic elements, we conducted a comparative spectral power analysis across seven frequency bands, averaging the results of all subjects, during passive virtual reality viewing of three distinct environments, with and without biophilic elements (Figure 2). There was a common pattern of the low- and the high-frequency power distribution across all three environments studied. In the lower-frequency bands (delta, theta, alpha), spectral power was the highest in the occipital, central, and frontal lobes, while the parietal and temporal regions exhibited lower activity levels. In the higher-frequency bands (beta, gamma), power was relatively higher in the occipital, temporal, and frontal lobes but lower in the central, parietal, and anterior prefrontal regions.

**Figure 3:**
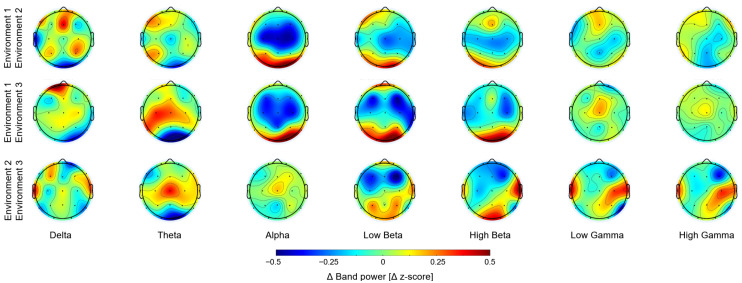
Comparison of band power differences between environments. The power in different frequency bands was compared by subtracting the values between environments. Similar differences were observed when comparing Env1 (non-biophilic) with Env2 and Env3 (biophilic).

To effectively visualize the differences in brain activity between the environments, the subtraction of band powers between environments were computed (Figure 3). The differences between Env1 (non-biophilic) and Env2, as well as between Env1 and Env3, showed a similar pattern across frequency bands. In the delta band, differences were observed primarily in the occipital and frontal lobes. In the alpha and beta bands, greater differences were found in the central and occipital lobes. In the gamma band, smaller differences were observed in the frontal, central, and occipital lobes. These findings indicate general brain regions where differences in the EEG spectral activity were localized between the environments with and without a biophilic element across various frequency bands.

**Figure 4:**
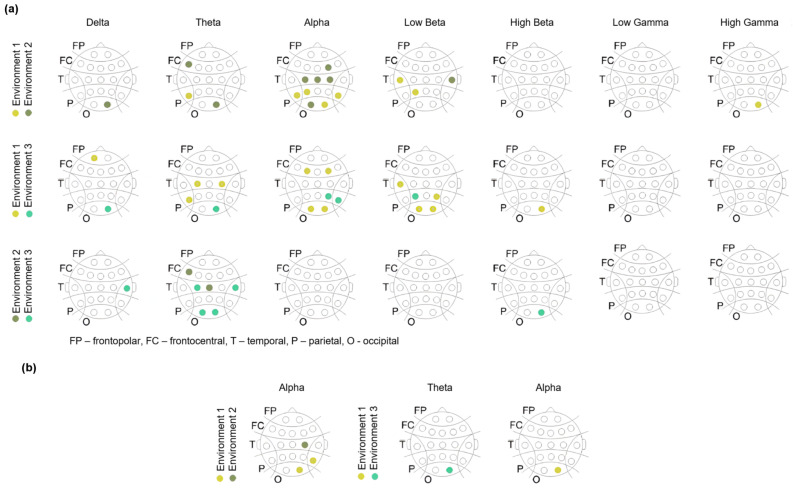
Alpha and beta spectral power in visual and parietal areas reflects responses to environments with biophilic elements. (**a**) Maps show electrode locations where a significant difference was found between environmental transitions (p<0.05). Columns represent frequency bands, while rows correspond to different environment comparisons based on Wilcoxon signed rank tests across all subjects. Two clusters of alpha and beta power responses emerge in the posterior visual areas. Highlighted electrode positions indicate significantly higher spectral power between specific environment pairs (color-coded). (**b**) The right occipital lobe maintained significance in the alpha band after Bonferroni correction.

Environments containing biophilic elements showed significant differences compared to the non-biophilic environment in the theta, alpha, and beta frequency bands, especially in the posterior visual areas of the occipital and parietal cortices (Figure 4a; see Table A1, Table A2 and Table A3 for electrode-specific *p*-values). We summarized all significant differences based on their polarity (higher or lower power), location, and frequency band at the level of specific EEG electrode contacts (Figure 4). In general, the environment without biophilic elements exhibited significantly higher alpha and beta spectral power at electrode locations within the identified posterior areas of interest. The alpha and beta power responses in the visual areas were consistently observed across the same group of electrode contacts, whereas responses in other frequency bands and regions were limited to individual electrode contacts. To ensure that these findings were not due to chance of multiple comparisons, Bonferroni correction was applied, which preserved the significant difference in occipital alpha power for Env1 when compared to Env2 and Env3 (Figure 4b).

**Figure 5:**
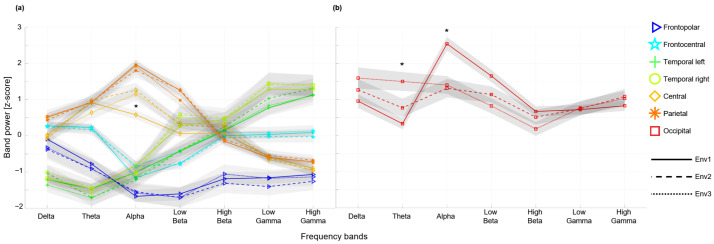
Introduction of biophilic elements selectively suppresses alpha and induces theta spectral activities in the occipital cortex. (**a**) Summary of the mean power-in-band averaged across electrodes in the same area channels reveals characteristic patterns for the posterior, central, temporal, and frontal lobes in response to viewing the studied environments. (**b**) The greatest response was observed selectively in the visual areas of the occipital cortex with a significant suppression of the alpha and an induction of the theta EEG activities. (* indicates p<0.05.)

Finally, we averaged the relative band power of all the electrodes in each of the seven areas to compare the identified alpha responses with other frequency ranges of the EEG spectrum. This activity revealed consistent patterns across cortical areas and the frequency bands (Figure 5a); the temporal areas in both hemispheres showed less power in the low-frequency bands (delta, theta) with gradually more power at higher frequencies. An opposite pattern of more power in the low-frequency bands, with gradually less power at high frequencies, was observed in the parietal and central areas. A similar pattern of higher power in the low-frequency bands dropping in the alpha, beta, and higher frequencies was consistent among the anterior frontopolar and frontocentral areas; the occipital areas were characterized by a relatively higher power across the entire spectrum (Figure 5b).

These differences are consistent with electrodes detected in statistical tests (Figure 4a,b). The identified posterior alpha power was the highest across all other EEG activities and brain regions. Switching between environments with and without a biophilic element induced the largest differences selectively in the occipital alpha activity (Figure 5b), revealing a significant suppression of power by introducing a biophilic element. Specifically, alpha activity was reduced at the O2 channel for Env1 compared to Env2 (Wilcoxon signed rank test, w = 227, z = 3.2628, *p* = 0.001) and for Env1 compared to Env3 (Wilcoxon signed rank test, w = 241, z = 3.7173, *p* = 0.0002). A similar suppression was observed in the central region at electrode channel C4, where alpha activity decreased significantly between Env1 and Env2 (Wilcoxon signed rank test, w = 25, z = −3.2953, *p* = 0.001). Apart from this main effect, there was also a significantly greater power in the occipital theta activity induced by introducing the biophilic elements. The theta power was higher at the same O2 channel for Env1 compared to Env2 (Wilcoxon signed rank test, w = 59, z = −2.1914, *p* = 0.0284) and for Env1 compared to Env3 (Wilcoxon signed rank test, w = 14, z = −3.6524, *p* = 0.0003). No other effects of introducing biophilic elements were observed in any frequency band or cortical area (Figure 5a). Hence, we associate the suppression of posterior alpha and induction of occipital theta EEG activities at a specific electrode channel with a visual response to the biophilic elements introduced in the virtual environment.

## 4. Discussion

Our study identified neural signatures of exposure to distinct environments in architectural spaces using immersive VR and EEG. By reducing the number of design features to a minimal set, we isolated the effects of the added elements on cortical activity and found that brief exposure to them produced two consistent neural responses: suppression of occipital alpha power and enhancement of occipital theta power.

The observed suppression of alpha activity in posterior cortices likely reflects increased sensory processing and attentional engagement. Alpha oscillations are widely considered to be inhibitory gating rhythms that regulate information flow, with lower alpha indicating greater cortical excitability and more active visual processing [43,44,45,46,47,48]. In our paradigm, natural features may have captured attention in an involuntary but effortless manner, consistent with Attention Restoration Theory [15,21]. The fact that alpha suppression was localized to occipital regions further supports the interpretation that these effects are driven by visual rather than general arousal mechanisms.

At the same time, the introduction of natural elements enhanced occipital theta activity. Theta rhythms are associated with the perception of novel or emotionally salient stimuli, as well as with relaxation and meditative states. This dual role aligns well with Stress Reduction Theory, which proposes that exposure to nature lowers stress and promotes positive affect [13,16]. Together, the suppression of alpha and the induction of theta point to a coordinated modulation of inhibitory and excitatory rhythms in visual networks. Previous studies have shown modulation activity in alpha and theta during exposures to nature [39,49,50].

The results suggest that neural oscillations can serve as objective biomarkers for assessing the impact of architectural elements. Posterior alpha suppression and theta enhancement may provide early indicators of how environments influence visual engagement and emotional state. This has direct applications in evidence-based design: architects and urban planners could test configurations of greenery in VR and evaluate their cognitive and affective impact before construction. In healthcare settings, integrating natural views or plant-based design features could support stress reduction and recovery, consistent with classic studies on patient outcomes [11,12].

Moreover, the paradigm used here highlights the value of immersive VR combined with EEGs for prototyping. Unlike static questionnaires or recovery measures, this approach enables controlled manipulation of design variables while directly capturing neural responses. Future studies could expand on this by incorporating other sensory modalities (sound, scent, airflow) that often accompany real-world exposure to nature and by testing longer-term exposures to better evaluate chronic restorative effects.

The current results do not provide evidence to support the notion that the effects of biophilic elements might vary depending on their scale and spatial integration within the virtual environment. The observed EEG responses were consistently associated with the presence versus absence of biophilic elements, without a clear differentiation between the two biophilic environments (Env2 and Env3). This suggests that, at least within the parameters of the environments tested, the mere presence of green elements is sufficient to elicit measurable changes in visual cortical activity, while variations in their scale or integration do not appear to produce additional or graded neurophysiological effects. It is possible that the spatial differences between Env2 and Env3 were not large enough to generate detectable EEG distinctions or that the neural markers measured here are primarily sensitive to qualitative presence rather than quantitative differences. Future studies incorporating more systematically varied scales and arrangements of biophilic elements may clarify whether these factors influence the neural responses.

Several limitations should be noted. First, our study compared environments with and without natural elements but did not include additional non-biophilic features as controls. Therefore, some of the observed neural and cognitive responses may have been influenced by novelty effects or other uncontrolled environmental variables, rather than reflecting the intrinsic properties of natural stimuli alone. Second, the brief exposure windows of 10 s were sufficient to capture immediate neural responses but are unlikely to reflect longer-term restorative or affective effects associated with prolonged interaction with biophilic environments. Moreover, the total experimental session duration of 7.4 min, while relatively short, may have contributed to attention drift or participant fatigue, as maintaining sustained engagement over several minutes without variation can reduce focus and modulate EEG measures. Third, our VR environments lacked the multisensory richness of real-world settings, and participants remained stationary, limiting ecological validity. Future studies should explore diverse biophilic configurations, incorporate multimodal cues, and allow interaction or exploration of environments. Finally, to gain a deeper understanding of the underlying brain processes, more sophisticated and robust feature extraction techniques could be implemented. While the present work relies on conventional frequency band power analysis, future studies could extend the analysis to time–frequency decompositions and nonlinear signal processing methods to better capture the spatiotemporal dynamics of EEG activity [51,52,53,54].

## 5. Conclusions

This study shows that brief exposure to biophilic architectural features in immersive VR elicits distinct neural responses, i.e., suppressed occipital alpha and enhanced theta activity, indicating increased visual engagement and affective modulation. By combining EEG with VR, this study demonstrates a robust framework for evidence-based architectural design, enabling an objective assessment of how environments influence cognition and emotion.

Although no differences were observed between the scales of biophilic integration, future research should explore broader design variations, longer exposures, and multimodal cues to enhance ecological validity. These findings also open promising avenues for integrating neurophysiological data into architecture and urban design. Brain–Computer Interface (BCI) technologies could enable spaces to adapt in real time to users’ cognitive and emotional states, optimizing comfort and engagement. Beyond architecture, such adaptive environments may support rehabilitation and therapy by tailoring sensory inputs to reinforce restorative brain states.

Advancing this line of research will require the identification of reliable neural markers for environmental adaptation and evaluation of the long-term outcomes of such interventions. Ultimately, neuroarchitecture has the potential to create dynamic, human-centered environments that bridge neuroscience, design, and technology.

## Data Availability

The raw data supporting the conclusions of this article will be made available by the authors upon request.

## Appendix A. Supplementary Tables

**Table A1:** Summary of significant difference *p*-values found between Env1 and Env2. Notice that the O2 channel showed significant differences across multiple frequency bands, and that the alpha band showed the greatest number of significant instances.

Freq. Bands	Channels									
Delta						O2 0.013				
Theta						O2 0.028	F7 0.033	T5 0.024		
Alpha	F4 0.005	C3 0.007	C4 0.001	P3 0.026	O1 0.008	O2 0.001	F8 0.036	T5 0.018	T6 0.002	CZ 0.003
Low Beta				P3 0.013				T3 0.036	T4 0.039	
High Gamma						O2 0.046				

**Table A2:** Summary of significant difference *p*-values found between Env1 and Env3. The O2 channel presented significant differences across multiple frequency bands, and the alpha band showed the greatest number of significant instances.

Freq. Bands	Channels					
Delta	FP1 0.011				O2 0.006	
Theta		C3 0.026	C4 0.049		O2 0.0002	T5 0.0424
Alpha	F3 0.031	F4 0.039	P4 0.028	O1 0.028	O2 0.0002	T6 0.024
Low Beta		P3 0.006	P4 0.008	O1 0.022	O2 0.003	T3 0.0495
High Beta					O2 0.039	

**Table A3:** Summary of significant difference *p*-values found between Env2 and Env3. The theta band exhibited multiple electrodes showing significant activity, including both O1 and O2.

Freq. Bands	Channels					
Delta					T4 0.039	
Theta	C3 0.031	O1 0.031	O2 0.018	F7 0.045	T4 0.033	CZ 0.049
High Beta			O2 0.039			
